# A compact time reversal emitter-receiver based on a leaky random cavity

**DOI:** 10.1038/srep36096

**Published:** 2016-11-04

**Authors:** Trung-Dung Luong, Thomas Hies, Claus-Dieter Ohl

**Affiliations:** 1 Division of Physics and Applied Physics, School of Physical and Mathematical Sciences, Nanyang Technological University, 21 Nanyang Link, 637371, Singapore; 2 DHI Water & Environment (S) Pte Ltd, 1 Cleantech View, 637141, Singapore

## Abstract

Time reversal acoustics (TRA) has gained widespread applications for communication and measurements. In general, a scattering medium in combination with multiple transducers is needed to achieve a sufficiently large acoustical aperture. In this paper, we report an implementation for a cost-effective and compact time reversal emitter-receiver driven by a single piezoelectric element. It is based on a leaky cavity with random 3-dimensional printed surfaces. The random surfaces greatly increase the spatio-temporal focusing quality as compared to flat surfaces and allow the focus of an acoustic beam to be steered over an angle of 41°. We also demonstrate its potential use as a scanner by embedding a receiver to detect an object from its backscatter without moving the TRA emitter.

Time reversal acoustics (TRA) is a self-adaptive technique which is capable of focusing an acoustic wave effectively through a lossless heterogeneous medium^1^,^2^,^3^. Compared to the traditional phase array method for signal focusing, TRA techniques offer the advantage of greatly reduced complexity for signal generation. One method to operate TRA is to launch an outgoing acoustic wave into the medium by applying an electrical pulse to a piezoelectric transducer, which we name the emitting transducer. During propagation through the medium, this initial acoustic pulse is refracted, reverberated and scattered, and is then recorded at a point of interest by a receiver converting the acoustic signal into an electrical signal. This electrical signal is stored, time reversed and fed to the emitting transducer. A time reversed wave is launched, which while propagating back into the same medium is focused to the place spatially and temporally where it was launched^4^. The time-invariance and the spatial reciprocity properties of the linear acoustic wave equation is responsible for this remarkable feature. TRA has been employed successfully for example in the fields of telecommunication^5^,^6^, medical ultrasound^7^, ocean acoustics^8^,^9^, nondestructive testing^10^,^11^,^12^, and impact sensing^13^.

Interestingly, a scattering medium along the wave propagation improves the focusing quality^2^ of TRA, in particular for systems with a small number of transducers. Montaldo *et al*.^14^ utilized the scattering within a solid metallic wave guide to generate high pressure waves for the fragmentation of renal stones in extra-corporeal shock wave lithotripsy. Here, the cylinder had a diameter of 32 mm and a length of 0.5 m. The system was able to deliver a maximum spatio-temporal pressure of up to 650 bar from only 7 TRA transducers glued to one end. Besides its use as a scattering waveguide, a smaller time reversal emitter termed kaleidoscope has been implemented^15^,^16^. It is a rectangular metal block with a quarter sphere being cut off from one of its corners. To focus the kaleidoscope in 3D it is driven by 30 transducers.

Sarvazyan *et al*.^17^ proposed a leaky cavity which is made of a crumpled plastic bottle filled with water and partially suspended in air. The cavity was coupled to the measurement tank through a small contact area. It was able to generate several foci and shift them off the main axis revealing little side lobes. Arnal *et al*.^18^ demonstrated a compact version (14 cm × 14 cm × 25 cm) of a scattering medium for lithotripsy applications, which is based on the idea of a leaky liquid filled cavity but now containing multiple scattering rods embedded. They obtained the high pressures needed by exciting the cavity with an array of 128 individually controlled piezoelectric elements.

Ideally a TRA based transducer system should be compact and obtain good focusing already with a small number of transducers while also possessing adequate focusing quality and steering capability. However, the above-mentioned methods use large numbers of transducers and bulky geometries, making them less attractive for TRA in medicine or for non-destructive diagnostics.

In this paper, we present a compact design suitable as an emitter and emitter-receiver device. Although its shape resembles that of acoustic diffusers used in room acoustics, this design operates partly also in transmission mode. They consist of closely packed metal pillars having random heights. Each pillar has a square cross-section and all are on top of rectangular shape platform, see Fig. 1a. We call this random surfaces “terrain structures”. Results from terrain structures with pillars of two different cross-sections are presented below.

A *TRA transmitter* is constructed from two terrain structures facing each other as shown in Fig. 1b and filled with water. Thereby a leaky cavity if formed, which is driven by one or several piezoelectric ceramics attached to the flat back of one of the two diffusers. The surface which the piezoelectric ceramics are attached is defined as the back of the cavity. We characterize the design by measuring the spatial pressure distribution, and the temporal and spatial focusing properties in TRA operation.

The TRA transmitter can be combined with a sensing transducer to form a *TRA-transmitter/receiver pair*. TRA transmitter-receiver is formed from two printed acoustic diffusers with piezoelectric ceramics attached to the flat back of one of the two diffusers which defines the back of the cavity. Through the central hole of the printed acoustic diffuser the sensing transducer is inserted. The surface of the sensing transducer is set flush with the flat surface of the other diffuser, see Fig. 1c. The cross-section of the *TRA-transmitter/receiver* is shown in Fig. 1d.

The present work is organized as follows: first we describe the design and construction of the TRA transmitter and transmitter/receiver and the experimental setup. Next, we investigate the ability of a single random terrain structure to generate a diffuse wave field. And then we move on with the TRA emitter composed of two terrain structures and study its temporal and spatial focusing property. At last we report on the scanning properties of the transmitter/receiver design to detect small reflectors.

## Material and Methods

### Fabrication of the acoustic diffuser

Our acoustic diffusers with the terrain structure are manufactured with a 3D metal printer. We first generate a volumetric model of the acoustic diffuser with a computational geometry programming language (OpenSCAD). The software allows to construct an object of primitives. Here the terrain structure consists of a support surface with height of 1 mm and area of 38 mm × 38 mm which is decorated with pillars of square cross-section (1 mm and 2 mm). Their height is generated randomly with values ranging between 0.5 mm and 10 mm distributed uniformly. The average thickness of the pillars in our design has the value of approximately 5 mm. Then the geometry is converted into a suitable format for the 3D metal printer (MLabcusing R, Concept Laser Gmbh). The printer uses a 100 W infrared fiber laser with a focus diameter of 40 μm to sinter austenitic stainless steel powder with an average grain size of 50 μm.

**Figure 1:**
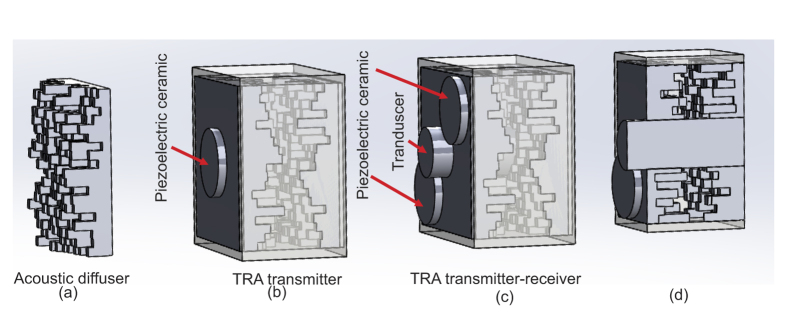
(**a**) Schematic drawing of 3d-printed acoustic diffuser constructed with multiple pillars of different length. (**b**) A TRA transmitter is formed from two printed acoustic diffusers facing each other. (**c**) TRA transmitter-receiver which is cut-open on the right and houses a pencil transducer in its center as a receiver. (**d**) The cross-section structure of the TRA transmitter-receiver.

From the diffuser plate, different designs of the TRA transmitter/receiver were derived, see Fig. 1. All have in common that 1 MHz piezoelectric discs (Steminc Inc, diameter of 2 cm) are glued to the flat back of the acoustic diffuser with a thin layer of epoxy. Silicone gel was applied to the other side of the piezoelectric disc for electrical insulation.

### Experimental setup

**Figure 2:**
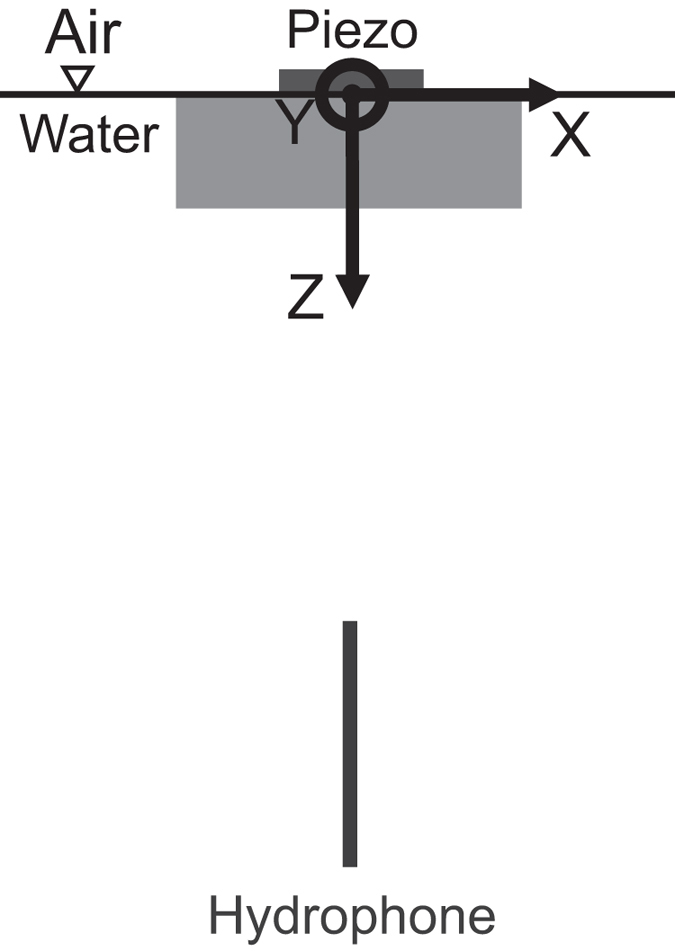
The acoustic diffuser (bright gray object) is submerged into the water such that only its back with the piezoelectric disc is in contact with air. The needle hydrophone is facing the diffuser. The origin of the coordinates is the water surface at the center of the piezoelectric disc (dark gray object).

The acoustic diffusers are mounted in a tight fit plastic holder, which allows inserting one or two of them as shown in Fig. 1. This housing is attached to a stainless steel rod and fixed in place at the free surface of a water filled basin made of transparent acrylic. By placing the piezoelectric transducer at the water surface, the back of the cavity becomes air-backed which increases the pressure amplitude transmitted into water, see Fig. 2.

The signal is generated with an arbitrary waveform generator (Agilent 33220A), fed to 45dB RF amplifier (350 MHz bandwidth, ENI 440LA) and connected directly to the piezoelectric element(s). For measurements of the spatial pressure distribution a single oscillation cycle at 1 MHz at 80 *V*_*pp*_ is applied to the piezo.

The water below the transducer in the basin is approximately 30 cm and is placed near the center of the 550 mm × 550 mm open surface area. The acoustic signals are measured with a small hydrophone (HNR-1000, ONDA Corporation) with a 1 mm diameter circular PVDF sensor. While the emitter/receiver is fixed to the center location we position the hydrophone with three programmable translation stages (Thorlabs, LTS 300). The coordinate system adapted in this manuscript fixes the origin at the center of the piezoelectric disc, the *x*- and *y*-coordinate are lateral to the water surface, and the *z*-axis increases with normal distance from the transducer. When scanning the sound field the hydrophone is translated in steps of 0.5 mm with *y* = 0, *x*  is varied between −100 mm and 100 mm and *z* = 150 mm.

The measurement has been automated during which the hydrophone is positioned, the electric signal is generated from a control computer, uploaded to the arbitrary waveform generator, triggered and then captured with a 12 bit sampling oscilloscope (LeCroyHD4340). The signals are then transferred to the computer and stored.

### Characterization of the acoustic diffusers

First, we wanted to characterize how diffuse the sound field is that is emitted from the terrain structured plates as compared to an ideal plane surface. The thickness of the flat plate is 5 mm, which is chosen to be similar with the average thickness of the diffuser.

**Figure 3:**
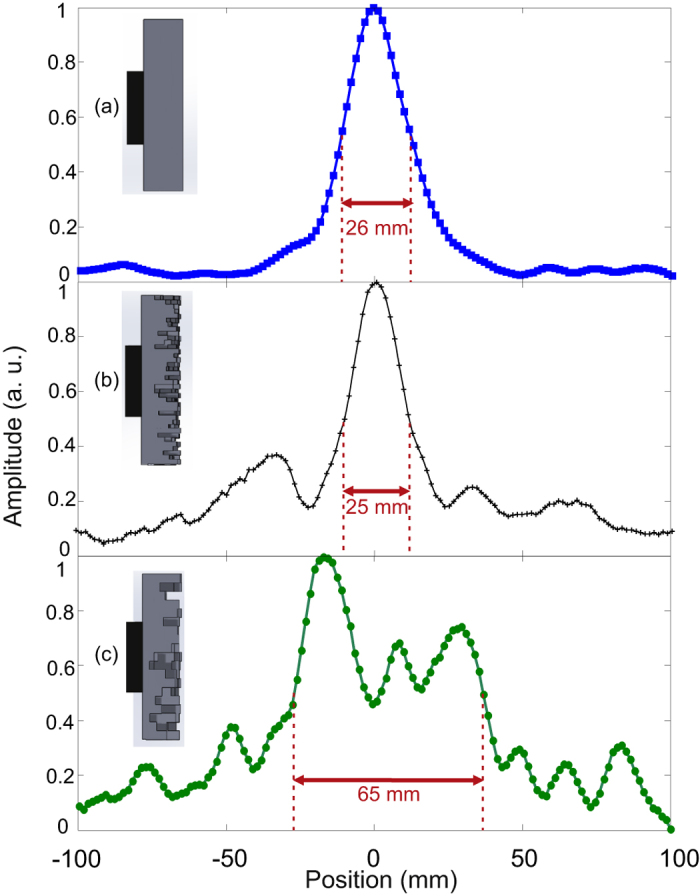
Spatial distribution of pressure amplitude from different structures: flat plate (**a**), terrain diffuser with 1 mm × 1 mm pillars (**b**) and terrain diffuser with 2 mm × 2 mm pillars (**c**). The spatial distribution of the flat plate and the 1 mm × 1 mm diffuser show one dominant peak while secondary peaks are found for the 2 mm × 2 mm diffuser.

Figure 3 shows the spatial distributions of the maximum positive pressure measured at a distance of 150 mm from the front side of the plate for three distinct configurations: flat plate (Fig. 3a), terrain structure with 1 mm pillars (Fig. 3b), and a terrain structure with 2 mm pillars (Fig. 3c). As expected, the flat plate provides a symmetric and narrow beam with a single distinct peak having a full width at half maximum (FWHM) of 26 mm. We observe a strong difference on the spatial pressure distribution using the terrain structure (1 mm pillar size). Several side lobes are prominently visible. Yet the FWHM of the main peak, approximately of 25 mm, has not significantly changed. The terrain with 2 mm pillar size alters greatly the shape of the central peak, it seems to consist of three peaks of approximately similar amplitude spanning around 65 mm at FWHM. This measurement suggests that the terrain diffuser with the wider pillars will perform better within the leaky cavity.

## Results and Discussions

To utilize the acoustic diffuser tested above for TRA, a reverberation cavity was constructed. To obtain a compact design we embed two parallel diffuser plates with the terrain surface facing each other. An ABS made plastic holder fixes and aligns the plates such that a gap of approximately 2.5 mm between the tallest pillars remains. Prior to assembly the diffusers were stored in a container of water, which was degassed in a vacuum chamber to prevent bubbles from being entrained between the pillars. They were then assembled into the cavity while remaining submerged. The final structure of the transducer, namely the pillar cavity, has dimensions of 40 mm × 40 mm × 22.5 mm. We compare the performance for TRA with a cavity formed by two undecorated plates of 5 mm thickness each (flat cavity). The same plastic holder is used to fix these plates. The gap between the two undecorated plates is roughly 12.5 mm.

The large mismatch in acoustic impedance between steel and water results in a high Q-value of the cavity, which is needed for the desired long reverberation times.

### Spatial pressure distribution of the cavity transducer

**Figure 4:**
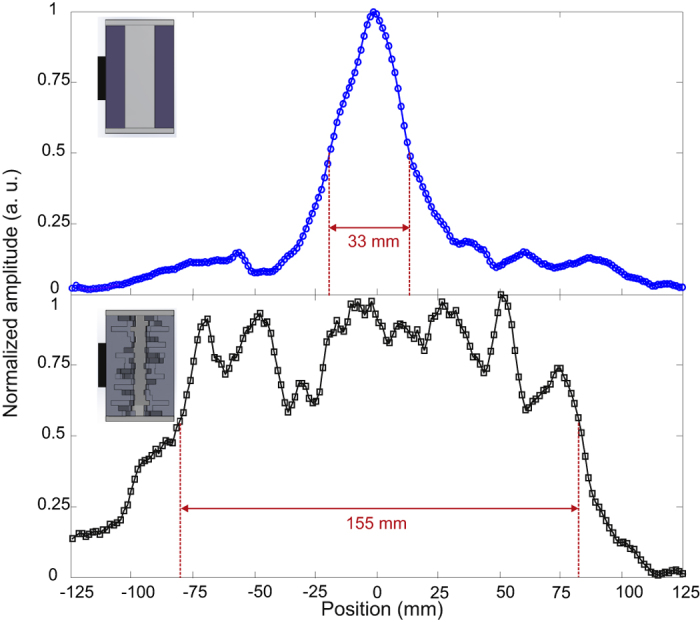
Distribution of the maximum pressure from (**a**) flat cavity and (**b**) terrain cavity with 2 mm × 2 mm pillars. Note the greatly enhanced FHWM of the central peak.

Before performing TRA, the pressure distribution from the cavities was measured to confirm a wide spatial emission. Here again a single cycle 1 MHz signal is used but the scan range is increased to −125 mm ≤*x* ≤ 125 mm and *z* = 150 mm of the hydrophone. The measured distribution of the maximum pressure emitted from the cavities is depicted in Fig. 4. The FWHM of the flat cavity Fig. 4a is about 33 mm while the terrain cavity in Fig. 4b displays a nearly 5-fold increased FWHM of 155 mm. We find that the cavity transducer with the terrain surfaces greatly enhances the spatial spreading while maintaining a compact form.

### TRA: Temporal focusing

**Figure 5:**
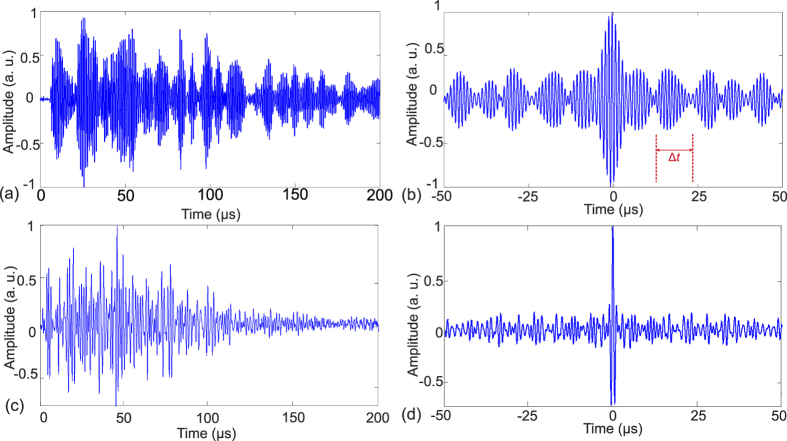
(**a**) Impulse response of the flat cavity measured with a hydrophone and (**b**) its TRA temporal signal. (**c**) Impulse response of the terrain cavity and (**d**) its TRA temporal signal. A very well temporal compression of the TRA signal is observed for the terrain cavity as shown by a distinct peak at time t = 0 s as compared to the flat cavity. Here, *t* = 0 s in b and d is aligned with the maximum of the TRA signal.

We next study the ability of the transducer to temporally focus a TRA signal. The TRA operation was performed at the location **r**(*x, y, z*) = (0, 0, 150) mm. Therefore, the tip of the needle hydrophone in our experimental setup was placed at **r** and records the signal from a single cycle 1 MHz sine wave. Because of the strongly reverberant cavity, the initial pulse is stretched into a long signal, see Fig. 5a,c. This signal was sampled with a 12-bit oscilloscope at a sampling rate of 2GSamples/s with an analog bandwidth of 350 MHz and transferred to a computer. A time window length of 200 μs of the stored signal, which contains the ballistic wave^19^, was selected. The window was then time reversed, loaded into the arbitrary waveform generator and amplified such that the maximum amplitude is the same as the one cycle input of 80 V_pp_.

Figure 5b,d depict the recorded signal after time-reversal. Both cavities lead to temporal compression of the TRA wave, see Fig. 5b,d. Yet, the terrain cavity delivered a considerably stronger temporal focus with a distinct peak shown in Fig. 5d. The smaller contrast for the flat cavity can be explained with a modulation of the emitted signal with a frequency of about 80 kHz (Δt~12.5 μs), visible in Fig. 5a,b. We expect that it is caused by the roundtrip time or free spectral range (FSR) of *f = c*/2*L*, where *c* = 1500 m/s is the speed of sound in water, and *L* = 12.5 mm the water gap between the two plates. The expected FSR is 60 kHz. In contrast the terrain cavity does not support a dominant mode and therefore shows a much better contrast.

### TRA: Spatial focusing

**Figure 6:**
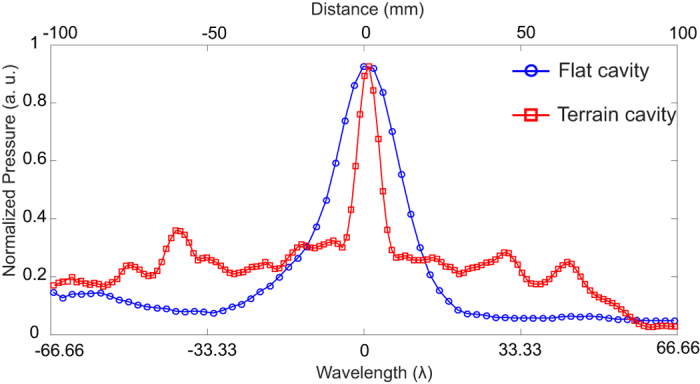
Spatial focusing quality of flat cavity (blue curve) and terrain cavity (red curve). The terrain cavity reveals a narrower focus.

Now we analyze the ability of the TRA operation to focus the acoustic signal spatially. The hydrophone was moved in steps of 0.5 mm along *x*-axis, with *z* = 150 mm and *y* = 0. For each position the maximum pressure is obtained and plotted in Fig. 6 for the flat and the terrain cavity transducer. The flat cavity results in rather poor focusing with a FWHM of 20 mm or ≈13 λ, while the terrain cavity achieves about 6 mm or ≈4 λ, where λ is the wavelength in water and has the value 1.5 mm for a frequency of 1 MHz. Theoretically^20^, the FWHM of a square aperture is approximately 1.2 λ*z*/*L*_S_, where *L*_S_ is the width of the square. In this experiment, the FWHM is predicted as approximately 4.5 λ for *L*_S_  ≈ 40 mm. The measured FWHM of the terrain cavity (≈4 λ) is close to the theoretically predicted FWHM. Although the spatial focusing is much better for the terrain cavity, we see a better contrast for the flat cavity.

### TRA: Steering of the spatial focus

**Figure 7:**
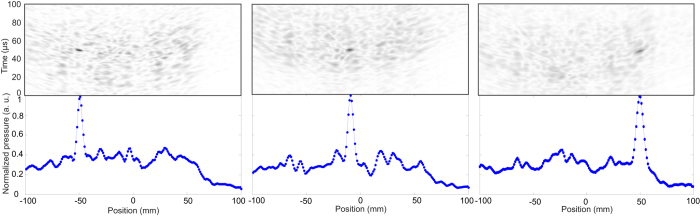
Top row: B-images with the spatial coordinate on the horizontal axis and time on the vertical axis to analyze the ability of steering the focus to the positions from left to right, *x* = −50, −10 and + 50 mm. Bottom row: Horizontal slice through the B-image at the time where the highest pressure is emitted revealing the spatial FWHM of the TRA focus. The peak pressures at *x* = −50, −10 and + 50 mm have the value of 10 kPa, 16.5 kPa, and 13 kPa, respectively.

After establishing the quality of the temporal TRA-signal we test the ability to steer the acoustic focus to different locations in the water bath. Therefore at selected locations along the *x*-axis the response to a 1 MHz single cycle was recorded and the TRA signal applied for each of these locations. These locations are *x* = −50 mm, −10 mm and + 50 mm with *y* = 0 mm and *z* = 150 mm. Then the TRA signal is applied to the transducer and the pressure as a function of time is measured along the *x*-axis from −100 mm ≤*x* ≤ 100 mm in steps of 0.5 mm, see Fig. 2. The results for the three different positions are shown in Fig. 7, where the horizontal axis is the *x*-axis and the vertical axis is time and the normalized pressure is color coded. This representation is called a B-image in ultrasound imaging^21^.The lower row in Fig. 7 depicts the pressure *p*(*x*, *t* = *t_max_*) at the time where the maximum pressure is measured, i.e. a horizontal slice in the B-image. These slices reveal that the FWHM of the focus for all three positions is approximately similar and again 6 mm wide, while having a signal to noise ratio (SNR) of about 6 dB. SNR here is calculated as 
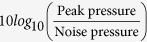

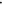
TRA at distances *x* > 60 mm (not shown in Fig. 7) still lead to similar FWHM but at a lower SNR. With the present design of a terrain cavity transducer we are able to obtain good focusing over a total lateral length of 120 mm at a distance of 150 mm away, corresponding to an opening angle of about 41°.

### Application as a scanner

Next we test if the TRA emitter-receiver can be used as an ultrasound scanner to detect acoustic reflectors. Therefore, we created a library of time-reversed signals in order to focus ultrasound at specific points in space. To generate this library, again one cycle of a 1 MHz signal was used to actuate the transducer. The emitted waves were recorded by the needle hydrophone at multiple positions in steps of 0.5 mm along the *x*-axis ranging from −100 mm ≤*x* ≤ 100 mm with *y* = 0 mm and *z* = 150 mm. We call this the scan line. The signals were then time reversed and stored in a computer. To perform the scanning operation, the corresponding time reversed files were subsequently called, loaded into the function generator, amplified and delivered to the transducer. The front surface receiver recorded all reflected signals. The position where a point reflector was located reflects the strongest signal.

**Figure 8:**
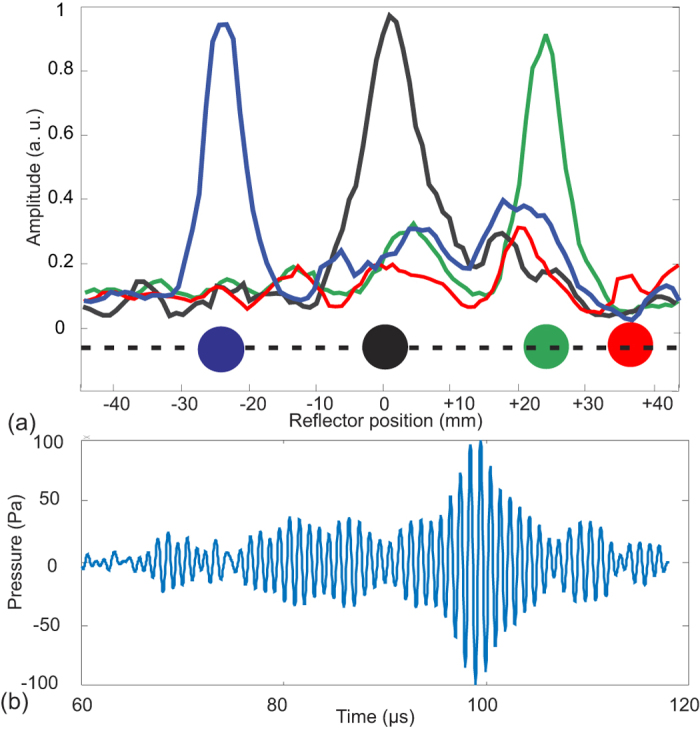
(**a**) Spatial scanning of point-like reflectors obtained with the emitter-receiver transducer. Peak of each signal corresponds to the location of the reflector. High SNR values were obtained when the reflector was positioned between −25 mm  < *x* < 25 mm. When the reflector was outside of this range, no distinct peak was detected (red curve). (**b**) The temporal received signal when the reflector is positioned at *x* = 0 mm.

In our experiment, a round split shot made of lead with a diameter of 6 mm was used as a point reflector. The reflector was attached to a nylon string with diameter of 0.25 mm and drawn along the scan line by two submerged fixtures. The background noise of the environment, S_n_, was recorded by the receiver before the ball was attached to the string. The receiver measured the reflected signals and the background noise S_n_ was subtracted. The reflected signals were further processed and plotted in Fig. 8. Normalized distinct peaks which corresponded to various positions are observed. Our TRA scanner was able to scan a point reflector at +/−25 mm where dominant peaks were still detectable. When the object was placed at position 35 mm no peak was detected, see the red curve in Fig. 8a. This is due to the fact that the pencil transducer we used to receive the acoustic echo has a limited beam width. To improve the length of the scan line, either a receiver with a wider aperture or more receivers would be required. Figure 8b shows the temporal measured signal when the reflector is positioned at *x* = 0 mm and the maximum signal is 0.1 kPa. The measured peak pressure when the reflectors are located at *x* = 25 mm is 0.06 kPa, and at *x* = −25 mm is 0.07 kPa, respectively. Nevertheless, this cost-effective TRA transducer which utilizes only a pair of transmitter and receiver has shown its applicability as a line scanner based only on the TRA effect thus without beamforming.

## Conclusion

In this paper, we have reported a compact emitter-receiver for time reversal acoustics. The emitter-receiver only required a small number of piezoelectric elements for operation. The acoustic diffuser which was employed in this emitter-receiver was manufactured by 3D printing technology. The emitter-receiver delivered using the terrain structures display spatio-temporal focusing much better than a simple design based on two flat plates. Additionally, the transducer can be steered over a wide angle of 41° and a TRA based ultrasound scanner was realized.

## Additional Information

**How to cite this article**: Luong, T.-D. *et al*. A compact time reversal emitter-receiver based on a leaky random cavity. *Sci. Rep.* **6**, 36096; doi: 10.1038/srep36096 (2016).

**Publisher’s note:** Springer Nature remains neutral with regard to jurisdictional claims in published maps and institutional affiliations.

## Supplementary Material

Supplementary Information
