# Supplementary material for: A compact time reversal emitter-receiver based on a leaky random cavity
Source: Sci Rep. 2016 Nov 4;6:36096. doi: 10.1038/srep36096 (PMC5095765; doi:10.1038/srep36096)
Supplement: Supplementary Information [file srep36096-s1.pdf]

Supplementary Information

Title: A compact time reversal emitter-receiver based on a leaky random cavity.

Authors: Trung-Dung Luong, Thomas Hies, Claus-Dieter Ohl.

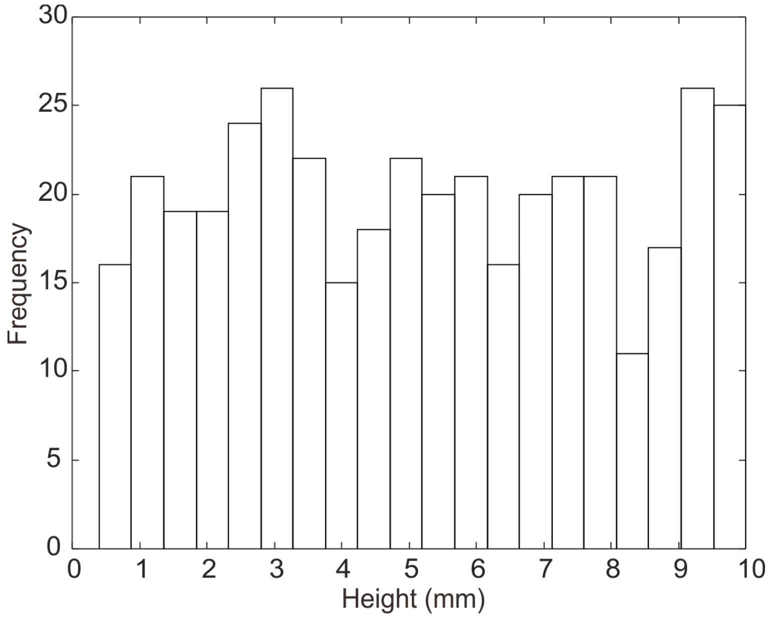

Figure S1: Histogram of the 2 mm pillars height of the terrain structure.

The height of the rods was generated using a pseudo-random number generator (frands) which comes with the standard library (stdlib) for the C++ programming language and is seeded with a constant value. The pseudo-randomness of the numbers produced by this library is very well-validated.

To be more quantitative a histogram of the 2 mm pillars height of the terrain structure is plotted in Supplementary Fig. S1.

In addition to Figure 3, the normalized pressure distribution of a terrain structure with 0.5 mm x 0.5 mm pillars is investigated and shown in Supplementary Fig. S2. Only a dominant peak is shown in the graph. The FWHM of the spatial distribution is 24.5 mm which is similar to the valued achieved in the flat plate and the 1 mm x 1 mm pillar structure.

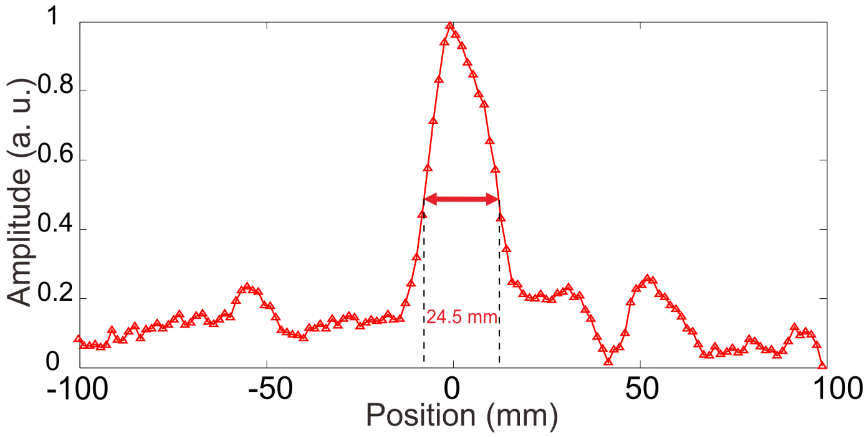

Figure S2: Pressure spatial distribution of a terrain structure with 0.5 mm x 0.5 mm pillars.
